# Keyword Index

**DOI:** 10.1038/sj.bjc.6604139

**Published:** 2007-12-11

1p 1416

3-phosphoinositide-dependent protein kinase-1 1465 3D tumour model 194

5-fluorouracil 334, 910, 1028, 1532 5-FU 919

adaptive conjoint analysis 717 adenomatous polyposis coli 384 adenosarcoma 1194 adenovirus 992 adjuvant chemotherapy 1021 adjuvant therapy 654 adjuvant; neutropaenia 1642 adolescent 29 adrenal tumour 420 adult T-cell leukaemia 1099 advanced cancer 177, 1475 advanced ovarian cancer 1200 aerobic glycolysis 646 aetiology 1287, 1291 Affymetrix 1165

ageing 1189 AKT 218, 785, 809 albumin 1266 alcohol 118 alcohol consumption 1287 alcohol intake 700 alcoholic beverages 429 alkaline sphingomyelinase 1441 ALL 992 allelic imbalance 1157 AML 877 anastrozole 152 androgen-independent 1206 androgens 688 androstene neuro-steroids 619 aneuploidy 1218 angiogenesis 391, 513, 1277, 1432, 1523, 1673 angiopoietin family 877 anilinoquinazolines 183 anti-adhesion 730 anti-EGFR treatment options 92 anti-folates 1071 antiangiogenic 65 anticancer agent 1344 antigen shedding 1146 antihypertensive treatment 112 antimetabolites 628 anxiety 1625 APC antibody specificity 384 apoptosis 253, 277, 637, 769, 902, 1077, 1225, 1234, 1432, 1532, 1696 aromatase 755 aromatase inhibitor 152 array-CGH 260 asbestos 1300 aspirin 1295 attributable proportion 129 audiovisual patient information 705 Aurora-A 1664 autologous transplantation 539 awareness 691 AZD2171 65

B-CLL 769 basement membrane 1505 BCG-refractory 1499 Bfl-1 769 biliary tract cancer 1577 biochemical recurrence 678 biomarker 1, 1457 biopsy 952 bladder cancer 1499 blood outgrowth endothelial cell 513 blood pressure 112 blood testing 1399 blood–brain barrier 322 body mass 1486 body mass index 995, 1005 bone metastasis 964 bone tumours 1588 bone turnover 964 bortezomib 1099 brain tumour 1583 *BRCA2* gene 826 breast 1642, 1673 breast and bladder cancer 502 breast cancer 58, 105, 152, 327, 434, 440, 479, 654, 686, 688, 725, 761, 809, 832, 957, 964, 978, 1046, 1090, 1157, 1175, 1211, 1251, 1277, 1287, 1361, 1372, 1505, 1523, 1570, 1625, 1632 breast cancer susceptibility 1696 breast conservation 883 breast surgery 1211 breast-conserving surgery (BCS) 725 breast-conserving treatment 1046

[^11^C]carvedilol 322 c-Kit 857 c-MET 368 C-reactive protein 1266 C4.4A 1146 C6 cells 755 Ca^2+^-binding protein 792

cancer clinical trials 705 cancer mortality 1300 cancer services 1588 cancer survivors 612 cancer survivorship 1625 cancer testis antigen 543 cancer vaccine 1381 capecitabine 712, 862, 1333, 1475 carbonic anhydrases 1465 carboplatin 162 carcinogenesis 646 carcinoma of an unknown primitive 857 cardiotoxicity 1084 case–control study 429, 434, 995 caspases 1234 CCR7 670 CD9 941 CDK4 1242 CEA 745 celecoxib 1206 cell cycle 277, 1513 cell cycle checkpoints 1664 cell death 1381 cell migration 1361, 1432 cell-free DNA 1399 cell-of-origin subtypes 1165 central nervous system tumours 1588 cervical cancer 129, 133, 218, 1322, 1457 cervical carcinoma 1058 cervix carcinoma 1135 CESH 1707 cetuximab 494, 1139 CGH 238 chemokine receptor 670 chemoprevention 327 chemoradiotherapy 712, 883 chemoresistance 769 chemotherapy 29, 862, 1028, 1040, 1157, 1234, 1642 Chernobyl 818 childhood 1583 childhood acute lymphoblastic leukaemia 140 childhood cancer 412, 695, 986 childhood leukaemia 986, 1009, 1315 Chile 85 China 123, 1577 *Chlamydia* 949 cholecystitis 1577 chromosomal instability 1218 chromosome 1696, 1707 chronic lymphocytic leukaemia 253 cigarette smoking 700 circumferential resection margin 1333 cisplatin 162, 194, 712, 851, 1234, 1329 clear cell adenocarcinoma 1053 clear cell renal cell carcinoma 420 clinical decision support systems 486 clinical phase II study 1084 clonality 1260 coffee 426, 1291 cognitive behaviour therapy 612 cohort 686, 1295, 1570 cohort studies 115, 118, 123, 129, 426, 1005, 1291, 1300 collagen I 358 colon cancer 1021, 1655 colorectal 971 colorectal adenoma 1425, 1449 colorectal cancer 297, 446, 745, 978, 1028, 1035, 1139, 1146, 1266, 1305, 1425, 1486, 1606 colorectal cancer cells 384 colorectal neoplasms 118, 1393, 1601 colorectal tumours 92 combination index 628 combination treatments 888 communication skills 472 comparative genomic hybridisation 1707 computer simulation 1322 conformal radiation 464 convection 194 copper transporter ATP7A 334 cost-effectiveness analysis 152, 1322 cost-utility analysis 152 Costa Rica 837 costs 479 COX-2 inhibitor 1206 COX2 557 coxibs 1465 CRH 637 CS-682 628 CXCL12 761 CXCR4 761 cyclooxygenase-2 1388, 1523 cyclooxygenase-2 independence 1465 cyclophosphamide 1200 CYP3A4 metabolism 440 cytokines 105, 210 cytotoxic T lymphocyte 1648, 1655

DDS 170 deep-vein thrombosis 1053 degradome 201 degranulating CD8^+^ cells 1381 dendritic cells 1251 depression 1625 deprivation 999 detection methods 1632 diabetes 995 Dickkopf-1 964 dietary iron 118 diffusion 194 disease progression 1469 disease-free survival 50 Dkk-1 1552 DNA crosslinking 253, 927 DNA damage 1189 DNA hypomethylation 412 DNA methylation 895, 1457 DNA repair 334, 927 docetaxel 290, 593, 851, 1206, 1338, 1475, 1613 doctor discipline 6 doctor specialty 6 dose intensity 1642 dose-limiting toxicity 177 downstaging 851 doxorubicin 43, 1084 doxorubicin resistance 1077 drug delivery 194, 735, 910 drug development 577 drug resistance 562, 927, 1218 ductal carcinoma *in situ* (DCIS) 725 dynamic models 1322 dysplasia 1545

E-cadherin 358 E2F 1242 early-onset disease 826 EBV 1567 EGFR 453, 494, 659, 778, 857, 1139, 1560 EGFR downstream signalling pathway 92 EGFR mutations 1560 elderly 162, 1606 ELISA 398 embryonic tumours 412 endometrial 1194 endometrial cancer 995, 1538

endoplasmic reticulum (ER) stress 1465 endoscopy 1493 endostatin 513 endovesical instillation 1499 England 989 ENPP7 1441 EpCAM (CD326) 315 epidemiology 112, 1287, 1583 epidermal growth factor receptor mutation 741 epidermoid cancer 1234 epigenetic process 1116 epigenetics 1, 267, 562, 986, 1416 epirubicin 1200 ErbB2 659, 1354 ErbB2 mutation 741 ErbB3 453 ESCC 1409 esophageal cancer 218 estramustine 1206, 1613 etoposide 162 expectations 14 extracellular matrix 201

α-fetoprotein 327 FACS 73 faecal occult blood 1601 familial 1175 Fas ligand 637 FASL 73 fatigue 612 FDG-PET 902, 1493 female 989 fetal origins of disease 686 fibroblasts 210, 398 first-line treatment 297 fluorescent *in situ* hybridisation 1139 foetal haemoglobin 412 folate 1449 folate metabolism 247 FOLFIRI 1035 follow-up 1632

G_1_ phase 1242 galectin-3 1146 gallstones 1577 GAMEC 308 gastric 902 gastric adenocarcinoma 458 gastric cancer 37, 123, 543, 550, 589, 700, 712, 837, 851, 1493, 1567 gastric cancer screening 837 gastric carcinoma 334 GDEPT 745 gefitinib 65, 183, 778, 1560 gemcitabine 43, 145, 283, 464, 1329, 1499 gene expression 145, 895 gene expression profiling 1432 gene fusion 1690 gene therapy 513 genetic alterations 260 genetic susceptibility 1701 genetics 1305 genomics 1116 germ cell 308 glioblastoma 302, 619 glioma cells 755 glucose transport 902 glucose-regulated protein 94 kDa 792 GLUT-1 646 glycosaminoglycan 761 glycosylation 1146 grapefruit intake 440 *GSTP1* 1399 Guthrie cards 992

haematological malignancies 1588 haeme oxygenase-1 1683 haplotype 832 HCC 1399 HCV 1399 HDAC 1344 HDAC inhibitor 177, 1344 head and neck cancer 65, 670 hemangiosarcoma 115 heme iron 118 heme oxygenase-1 1099 hepatic resection 1606 hepatitis C virus 426 hepatocellular carcinoma (HCC) 50, 426, 582, 862, 1260, 1532 HepG2 1441 HER dimerisation inhibitors 1338 HER-2 105, 494 Her-2/neu overexpression 857 HER2 453 HER3 453 hereditary 486 hetastatic breast cancer 1040 hexokinase 902 high dose 308 high-dose chemotherapy 391, 1200 high-risk breast cancer 391 histone modifications 1 histopathology 1393 HLA-A3 supertype 1648 HMEC 1361 Hodgkin lymphoma 29 Hodgkin's 1310 homocysteine 1071 hormonal 1194 hormone refractory 378 hormone replacement therapy 118, 1486 hormone-refractory 1480 hormones 688 HPV 218 HPV infection 133 HPV vaccination 1322 HRQOL scores 302 Hsp90 741 human 992, 1441 human gastric cancer 895 human papillomavirus 85, 691 hypercalcaemia 183 hypericin 502 hypermethylation 267 hypertension 995 hypomethylation 267 hypoxia 646

IBC signature 1165 Id proteins 1409 IL-6 378 IL-6R 378 imatinib 735 immune function 1251 immune privilege 637 immunohistochemistry 398, 405, 523, 531, 1135, 1404 immunostaining 952 immunotherapy 210, 315, 598, 1354 immunotherapy CD40 1251 impact evaluation 837 improvement 1469 incidence 1009, 1567 individual dosing 290 indomethacin 1077 infection 1310 inflammatory breast cancer 659, 883 inhibitor of apoptosis 1271 insulin 98 insulin-like growth factor 98 insulin-like growth factor binding protein 98 integration 85 integrins 1090 interferon resistance 231 interferon-*γ* 420 interleukin-6 1513 international differences 1277 interphase FISH 678 interstitial fluid pressure 735 intervention 612 invasion 73, 358, 368, 1505 invasiveness 50 ionising radiation 1664 irinotecan 297, 593, 1035 irradiaton 322 isolated tumour cells 589

JAK 378 JNJ-16241199 1344

K-Ras 1139 *K-ras/BRAF* mutations 1425 Ki-67 1124 KIF2C 543 kinase inhibition 741 knowledge 691, 705

*Lactobacillus rhamnosus* GG 1028 late complication 1058 Lck 1648 left-handedness 686 letrozole 802 leucovorin 297 leukocytes 978 lifestyle factors 133 ligand interactions 1361 liver cancer 1005 liver diseases 1441 liver metastases 1035 liver tumour 1441 Livin/ML-IAP/KIAP 1271 local recurrence 1046 locally advanced 464 locally advanced rectal cancer 1333 locoregional relapse 1632 long-term effect 612 loss of heterozygosity 1157 lung cancer 183, 247, 735, 1295, 1300 lung carcinoma 85 lymphadenectomy 1194 lymphoma 539, 619, 1310

malignant glioma 619 malignant melanoma 231 mammary carcinoma 1354 marker 1545 Masaoka's staging system 22 mass screening 1601 mast cells 952 maternal 688 mathematical models 646, 1322 matrikine 1505 matrix metalloprotease 1106 matrix metalloproteinase 398, 971, 1505 maximum tolerated dose 177 MCAK 543 MCM-2 1124 MCM-5 1124 MDA-MB-231 1361 medical education 472 medulloblastoma 267 melanoma 223, 1225, 1329 melatonin 755 mesenchymal stem cell 1552 mesothelioma 1300 meta-analysis 1005, 1291 metastases 868, 1606 metastasis 223, 670, 761, 957, 1409, 1523 metastatic gastric cancer 593 methotrexate 308 methylation 1425 microarray 818 microdose 577 micrometastasis 550 micronuclei 1218 microtubule 1218, 1673 microtubule inhibitors 888 microvessel density 391, 405, 1277 midkine 405 migration 543, 1090 minimum dataset 1393 mitochondria 345 mitogen-activated protein kinase 659 mitomycin 1475 mitotic catastrophe 941 mitoxantrone 1613 MMP 957 modulation 919 molar pregnancy 986 monoHER 1084 monohydroxyethylrutoside 1084 monounsaturated fat 1570 morphine 1523 mortalin 941 mortality 1009 mortality trends 1588 motility 358 MRP1 1077 MS-MLPA 1457 Ms-SNuPE 1457 MSC 1552 MSP 1457 MT1-MMP 358 MUC-1 730 MUC1 *O*-glycosylation 910 MUC4 345 mucin 345 multicell layer 194 multicentric occurrence 1260 multidrug resistance 502, 934 multifocal low-grade urothelial cancer 260 mutation 778, 1416 *Mycoplasma* 949

*N*-acetylglucosaminyltransferase V 1538 natural killer cells 539 neoadjuvant 1035 neoadjuvant chemoradiotherapy 1333 neoadjuvant chemotherapy 589 neoplastic syndromes 486 nested case–control study 426, 446 neuroblastoma 210, 238, 1416 neuron-glia-related cell-adhesion molecule 531 neuropilins 1090 neutropenia 37 NF-*κ*B 523, 745, 1165 NF-κB 659 NHE 646 NK cytotoxicity 105 NK105 170 non-small-cell lung cancer 283, 741 NSAIDs 557 NSCLC 1560 nuclear localisation 1683 nucleoside analogue 628 nucleoside diphosphate kinase 1372

obesity 1005 occupational exposure 1300 octreotide 582 oesophageal cancer 123, 494, 868 oesophagus 1404 oestrogen receptor 58, 659 offspring gender 688 oncogenes 1189 oncology 472, 1063 opioids 1523 OPN 1545 oral squamous cell carcinoma 792 osteonectin 1106 osteosarcoma 1552 outcome 1632 ovarian adenocarcinomas 1124 ovarian cancer 358, 637, 927, 1053, 1618 ovarian low malignant potential tumours 1124 ovarian neoplasms 1291 overall response rate 1618 overrepresentation analysis 1432 oxaliplatin 593, 862 oxaliplatin resistance 334 oxidative stress 1683

P-glycoprotein 322, 934 p16INK4A 1242 P2Y receptors 1372 p53 58, 1225, 1532, 1664 paclitaxel 43, 170, 888, 1218 palliative 283 palliative care 472 pancreas cancer 464 pancreatic adenocarcinoma 598, 1106 pancreatic cancer 98, 345, 910, 1432 pancreatic carcinoma 405, 523 pancreatic neoplasms 1116 pangenomic analysis 238 panitumumab 1469 papillary thyroid carcinoma 531 papillomavirus 129 papillomavirus, human 989 parental occupation 1315 passive smoking 434 pathology 654 pathway analysis 792 patient recruitment 705 patient-centred assessment 1063 patient-reported outcomes 1469 PBD dimer 253 PDK-1 785 pegylated interferon 1532 pemetrexed 1071 peptides 1648, 1655 performance status 458 pericellular proteolysis 201 peritoneal dissemination 550 pertuzumab 1338 PHA680632 1664 pharmacodynamic markers 1071 pharmacodynamics 290, 577 pharmacogenomics 145 pharmacokinetics 43, 290, 577 phase 0 trial 577 phase I 844, 1338 phase I study 170 phase I/II study 712 phase II clinical trial 862 phase II study 1480 phosphorylated Akt 92 phosphorylated MAPK 92 photochemical internalisation 502 photodynamic therapy 502, 1381, 1513 physical activity 995 PI3K/Akt 453 *PIK3CD* 1416 pineal 755 plasma 25-hydroxyvitamin D 446 plasma D-dimer 1053 plasma deoxynucleosides 1071 platelets 978 platinum chemotherapy 927 platinum-resistant 1618 platinum-sensitive 1618 PLD1 809 polymer micelles 170 polymorphisms 145, 557, 1175, 1449 polyphenolic compounds 1372 polyunsaturated fat 1570 poor prognosis 792 poor-risk 162 population analysis 290 population dynamics 140 population study 29, 112 population-based 730 population-based cohort study 986 positive lymph nodes 605 post-cancer fatigue 612 postmenopausal 1486 postnatal vasculogenesis 513 poverty 140 PPAR 1388 predicting factor 405 predisposition 1305 preeclampsia 688 premenopausal 802 prenatal infection 992 preoperative 802 primary colon cancer cells 73 primary health care 486 primary therapy 802 probiotic 1028 profilin 1361 prognosis 1, 37, 238, 247, 523, 550, 957, 1211, 1409, 1690 prognostic biomarker 678, 877 prognostic factors 302, 391, 605, 952, 1538 prognostic marker 730 programmed cell death 619 progression-free survival (PFS) 1538, 1618 proline-rich tyrosine kinase2 (Pyk2) 50 promoter 277 promoter hypermethylation 1260 prostate 378, 941, 1673 prostate cancer 557, 730, 826, 952, 978, 1206, 1480, 1613, 1683 prostatic neoplasms 1690 proteasome inhibitor 1099 protein-truncating *BRCA2* mutations 826 proteomics 1116 PSA 678 psychosocial 1063 PTEN 1139 *PTEN* haploinsufficiency 678 PTHrP 183 public 691 pulmonary MALT lymphoma 949 pulmonary thromboembolism 1053

quality of life 283, 1469, 1625 quantitative RT–PCR 531

R306465 1344 race 14, 140 radiation 1058, 1505 radiation effects 115 radiation susceptibility 818 radiosensitivity 1696 radiotherapy (RT) 65, 115, 725 randomised controlled trials 486 randomised trial 1200 rapamycin 809 Ras signalling pathway 1425 RASSF2 1425 real-time methylation specific PCR 1260 real-time PCR 1399 recurrence 50, 479 red meat 118 RelA 523 relative survival 999 renal cell cancer 112, 429 renal cell carcinoma 1271 resistance 919 review 1005 rhabdomyosarcoma 785 risk 971, 1486

S-1 37, 458, 851 S100 267 S6 kinase 218 sarcoma 115, 1194 saturated fat 1570 SCC 1545 SCLC 1077 Scotland 999 screen-detected 725 screening 129, 971, 1493 second cancer 1058 second soft tissue sarcoma 695 SEER-Medicare 1606 self-report 14 senescence 941, 1225 sensitivity 1493 sequential chemotherapy 1613 seroepidemiologic studies 989 serum 778, 978 sex 140 *SFRP*s 895 shared decision-making 6 signal transduction 368 single nucleotide polymorphism 832 siRNA 384 SJG-136 253 skin reactions 14 Smad-pathway 1388 small cell lung cancer 368 small molecular inhibitor 785 small molecule 1344 small-cell lung cancer 162 smoking 434, 1287 SNP 247 social contact 1315 social difficulties 1063 socioeconomic inequalities 999 socioeconomic status 140 SOCS proteins 231 solid tumours 539, 844 soluble interleukin-6 receptor 1513 somatostatin analogues 582 sorafenib 1480 spermatocytic seminoma 1707 splice 277 sporadic 1175 squamous cell carcinoma 1404 St Gallen expert consensus meeting 654 stage II 1021 staging 868, 1393 STAT 378 STAT phosphorylation 231 stealth liposome 919 stellate cells 1106 stem cells 1189 stereology 1135 stromal 1194 subsite 700 superoxide dismutase 2 1116 surgery 1690 survival 283, 523, 670, 1124, 1266, 1567 symptom 1469 synergy 253, 628 synthetic vectors 210

T-cell signalling proteins 105 tailor-made therapy 145 tamoxifen 152, 327, 582 tea 1291 teenage and young adult cancer 1588 telomere 832, 1696 temozolomide 1225 *TERF2* 832 *TERT* 832 testicular germ cell tumour 1701, 1707 testicular microlithiasis 1701 TGF-*α* 1388 TGF-*β* signal 1388 the Sloane Project 725 three weekly schedule 1040 thymic epithelial tumour (TET) 22 thymidine phosphorylase 745 thymidylate synthase 334, 1242, 1449 thymidylate synthase inhibition 1071 thyroid cancers 818 time-dependent variable 37 TIMP 957 tissue microarray 785 titanocene 1234 TMX2-28 cells 809 TNM stage 1266 toll-like receptor 598 tonsil 670 topoisomerase II 58 total dietary fat 1570 toxicity 577, 712 TP53 1157 trabectedin 1618 TRAIL 73 transcription 277 transformation 1457 transforming growth factor-*β* 398, 1106 transforming growth factor-*β* receptor 1 1175 trastuzumab 494 treatment 14 treatment decisions 6 treatment outcomes 717 treatment preference 717 treatment tradeoff method 717 trends 1009 treosulfan 1329 trichostatin A 562 trifunctional antibodies 315 tumour 277, 513 tumour angiogenesis 1372 tumour immunity 1655 tumour infiltrating lymphocytes 1135 tumour marker 412 tumour microenvironment 398, 1106 tumour therapy 1271 tumoural invasion 957 twenty year follow-up 730 tyrosine kinase inhibitor 65, 453 tyrosine kinase receptor 857

UFT 297 undergraduate 472 understanding 705 urbanisation 140 uterine cancer 605

valproic acid 177 vascular disrupting 888 vascular endothelial growth factor 391 VEGF 851 VEGF family 877 VEGF-C 1090 VEGF_165_b 223 VEGF_*xxx*_b 223 vinorelbine 283 viral load 85 vitamin D 123

weekly docetaxel 1040 weekly epirubicin 1040 weekly schedule 1040 western blot 531 white cells 1266 WHO histologic classification 22 Wnt signalling 964 women 1295 wound healing 1211

X-ray 1583 xanafide 58 XELOX 862 xenografts 73, 919 XR5944.14 844

young age 1046

ZD1839 183 ZD6126 888 ZD6474 934

